# Supplementary material for: Content-rich biological network constructed by mining PubMed abstracts
Source: BMC Bioinformatics. 2004 Oct 8;5:147. doi: 10.1186/1471-2105-5-147 (PMC528731; doi:10.1186/1471-2105-5-147)
Supplement: Additional File 2 — The original results of the above study (non-essential files are deleted to keep the file size under the limit set by BMC bioinformatics). [file 1471-2105-5-147-S2.bz2 › chilibotAdditionalFile2/dip50/427ID11470772E10357/html/CCL2_CCR2.html]

 


 **CCL2** and **CCR2** 
  
Found 2851 abstracts in PubMed, retrieved 50.  
 

 What does Google say? 
 PDF only 
| .edu only 

---

**Interactive relationship** (e.g. stimulation, inhibition, etc)

**Stimulatory relationship**- RESULTS Cell adhesion to fibronectin induced phosphorylation of FAK and MCP 1  [ **CCR2** ]   [ **CCL2** ]  mRNA expression in time and dose dependent manners followed by increased MCP 1  [ **CCR2** ]   [ **CCL2** ]  protein expression.  Ref: 12846738 Kidney Int, 2003
**Neutral relationship**

**Non-interactive relationship** (e.g. studied together, co-existance, homology, etc.)

- Cauterized corneas treated with anti MCP 1  [ **CCR2** ]   [ **CCL2** ]  antibody had significantly fewer monocytes macrophages than control.  Ref: 12861033 Lab Invest, 2003
- We found that in rabbit aortae, 1 high cholesterol diet feeding caused lipid deposition and intimal thickening, induced expression of P selectin, VCAM 1, MCP 1  [ **CCR2** ]   [ **CCL2** ]  and iNOS, and impaired acetylcholine ACh evoked vasorelaxation.  Ref: 12860246 Atherosclerosis, 2003
- Increased concentrations of cytokines, IL 6, IL 8, and MCP 1  [ **CCR2** ]   [ **CCL2** ] , were detected in the supernatant of cocultures of HUVEC with biomaterial treated whole blood or mononuclear cells but not neutrophils or platelets, compared with the media control.  Ref: 12870471 J Biomed Mater Res, 2003
- Concerning the other cytokines and chemokines studied MCP 1  [ **CCR2** ]   [ **CCL2** ] , IL 8 and IL 12, no significant differences were found to be associated with the radiological profiles.  Ref: 12872499 G Ital Med Lav ErgonG Ital Med Lav Ergon, 2003
- While  **CCL2**  inhibition in patients with acute respiratory distress syndrome ARDS may be hazardous by interfering with defense against bacteremia, future studies are needed to determine if  **CCL2**   **CCR2**  antagonism will offer breakthrough therapy for patients with allergic asthma, IPF, or BOS, and to confirm the hypothesis that  **CCL2**  polymorphism places patients at greater risk for these disorders.Microcirculation 2003 1 73 288.  Ref: 12851645 Microcirculation, 2003
- In vitro, macrophages from PAFR animals did not phagocytose trypomastigote forms when activated with PAF, leukotriene B 4 or MCP 1  [ **CCR2** ]   [ **CCL2** ]  and produced less nitric oxide when infected and activated with IFN gamma.  Ref: 12850205 Microbes Infect, 2003
- Despite intensive screening, there are as yet no small molecule antagonists of the receptor of MCP 1  [ **CCR2** ]   [ **CCL2** ]   **CCL2** ,  **CCR2** .  Ref: 12851642 Microcirculation, 2003
- Indeed, we find that RP3 alone causes increases in nuclear NF kappaB activity and secretion of MCP 1  [ **CCR2** ]   [ **CCL2** ]  and GM CSF.  Ref: 12881713 Oncogene, 2003
- Significant Involvement of  **CCL2**  MCP 1  [ **CCR2** ]   [ **CCL2** ]  in Inflammatory Disorders of the Lung.  Ref: 12851645 Microcirculation, 2003
- IL 6, MIP 2 and MCP 1  [ **CCR2** ]   [ **CCL2** ]  appear to follow the toxicity.  Ref: 12883092 Toxicol SciToxicol Sci, 2003
- In addition, MCP 1  [ **CCR2** ]   [ **CCL2** ]  production of PPD or 30 kDa antigen stimulated monocytes was significantly elevated in CR TB patients than that from E TB.  Ref: 12861395 Inflamm Res, 2003
- DESIGN AND PATIENTS Immunocytochemistry and reverse transcriptase polymerase chain reaction RT PCR amplification were used to analyse the expression of monocyte chemoattractant protein MCP 1  [ **CCR2** ]   [ **CCL2** ] , RANTES regulated upon activation, normal T cell expressed and secreted, macrophage inflammatory protein MIP 1alpha, MIP 1beta, interferon IFN gamma inducible protein IP 10 and monokine induced by IFN gamma Mig in thyroid tissue from patients with Hashimoto s thyroiditis n = 4, Graves disease n = 6 and nonautoimmune multinodular goitre n = 4.  Ref: 12864798 Clin Endocrinol (Oxf), 2003
- CONCLUSION Our results indicate that integrin mediated cell adhesion to the ECM can induce MCP 1  [ **CCR2** ]   [ **CCL2** ]  expression through activation of FAK, and suggest a role for altered ECM deposition in the progression of glomerular diseases by affecting gene expression.  Ref: 12846738 Kidney Int, 2003
- CONCLUSIONS The data suggest that MCP 1  [ **CCR2** ]   [ **CCL2** ]  production is not uniquely elevated systemically in TB patients, although MCP 1  [ **CCR2** ]   [ **CCL2** ]  production might be elevated by monocytes in the chronic phase of TB or with a local pleural infection.  Ref: 12861395 Inflamm Res, 2003
- Secondary lymphoid tissue chemokine SLC CCL21 on high endothelial venules triggers arrest of rolling lymphocytes, and keratinocyte derived chemokine KC mouse Gro alpha, CXCL1, monocyte chemoattractant protein 1  [ **CCL2** ]  MCP 1  [ **CCR2** ]   [ **CCL2** ]   **CCL2** , and regulated on activation, normal T cell exposed and secreted RANTES CCL5 trigger arrest of rolling monocytes.  Ref: 12851646 Microcirculation, 2003
- After 24 h, cytokine release was significantly increased for both IL 8 and MCP 1  [ **CCR2** ]   [ **CCL2** ]  but not IL 6 above concentrations after 4 h of coculture.  Ref: 12870471 J Biomed Mater Res, 2003
- ... transfection of dominant negative FAK abolished adhesion induced MCP 1  [ **CCR2** ]   [ **CCL2** ]  expression.  Ref: 12846738 Kidney Int, 2003
- METHODS MCP 1  [ **CCR2** ]   [ **CCL2** ]  mRNA and protein expression in cultured rat mesangial cells MC attached to ECM proteins were examined by reverse transcription RT polymerase chain reaction PCR and Western blotting, respectively.  Ref: 12846738 Kidney Int, 2003
- Monocyte chemoattractant protein MCP 1  [ **CCR2** ]   [ **CCL2** ]  and macrophage colony stimulating factor M CSF are two of the associated chemokines, but their role in coronary artery disease has not been sufficiently clarified.  Ref: 12892039 J Cardiol, 2003
- Pyrrolydine dithiocarbamate, an inhibitor of NF kappaB was also able to reduce the eotaxin, MCP 1  [ **CCR2** ]   [ **CCL2** ]  and MCP  [ **CCL2** ]  3 expression and production 50 13%, P <.  Ref: 12854631 Respir Med, 2003
- We found that clinically relevant levels of Hcy 10 to 1000 micro mol L increased the protein secretion and mRNA expression as well as activity of MCP 1  [ **CCR2** ]   [ **CCL2** ]  and IL 8 in cultured primary human monocytes.  Ref: 12881478 Circ ResCirc Res, 2003
- Five genes were expressed at lower levels in unstable angina samples anticoagulant protein S, cyclooxygenase COX 1, interleukin IL 7 and chemokines monocyte chemotactic protein MCP 1  [ **CCR2** ]   [ **CCL2** ]  and 2.  Ref: 12871422 J Thromb Haemost, 2003
- For monocytes, CR TB patients spontaneously expressed more MCP 1  [ **CCR2** ]   [ **CCL2** ] , compared with HTRs and E TB patients.  Ref: 12861395 Inflamm Res, 2003
- Groups of rats were exposed to hypoxic and hyperoxic environmental conditions for 60 h and MCP 1  [ **CCR2** ]   [ **CCL2** ]  was determined in their peripheral blood mononuclear cells by Elisa and Reverse Transcriptase Polymerase Chain Reaction RT PCR.  Ref: 12870648 Mol Cell Biochem, 2003
- The reduction in the lesion size was correlated with the reduced expression of MCP 1  [ **CCR2** ]   [ **CCL2** ]  mRNA and protein, as detected by real time quantitative polymerase chain reaction and immunohistochemistry respectively.  Ref: 12844369 Br J Nutr, 2003
- Whereas treatment with infectious virus induced a rapid down regulation of the  **CCL2**  monocyte chemoattractant protein 1  [ **CCL2** ]  MCP 1  [ **CCR2** ]   [ **CCL2** ]  specific receptor  **CCR2** , inactivated virus did not significantly alter  **CCR2**  surface expression.  Ref: 12885942 J Leukoc Biol, 2003
- Because MCP 1  [ **CCR2** ]   [ **CCL2** ]  induces monocyte immigration and differentiation to macrophages, which augment extracellular matrix production and tubulointerstitial fibrosis, pharmacological reduction of angiotensin II may also exert its beneficial effects in diabetic nephropathy by downregulation of renal MCP 1  [ **CCR2** ]   [ **CCL2** ] .  Ref: 12882873 Diabetes Care, 2003
- The MCP 1  [ **CCR2** ]   [ **CCL2** ]  levels were measured by enzyme linked immunosorbent assay ELISA.  Ref: 12861395 Inflamm Res, 2003
- and weak for IL 1beta and monocyte chemotactic peptide 1 MCP 1  [ **CCR2** ]   [ **CCL2** ] .  Ref: 12874304 Infect Immun, 2003
- ... IL 6, MIP 2 and MCP 1  [ **CCR2** ]   [ **CCL2** ]  were increased by 4 8 hours.  Ref: 12883092 Toxicol SciToxicol Sci, 2003
- This study investigated the expression of MCP 1  [ **CCR2** ]   [ **CCL2** ]  and c fms M CSF receptor in human coronary tissues.  Ref: 12892039 J Cardiol, 2003
- Plasma was assayed for endothelin 1 ET 1, asymmetric dimethylarginine ADMA, intercellular adhesion molecule 1, vascular cell adhesion molecule 1 VCAM 1, and monocyte chemoattractant protein 1  [ **CCL2** ]  MCP 1  [ **CCR2** ]   [ **CCL2** ] .  Ref: 12847693 Arthritis Rheum, 2003
- Monocyte chemotactic protein 1  [ **CCL2** ]  MCP 1  [ **CCR2** ]   [ **CCL2** ] , an important chemotactic cytokine can be activated by active oxygen species.  Ref: 12870648 Mol Cell Biochem, 2003
- Homocysteine stimulated MCP 1  [ **CCR2** ]   [ **CCL2** ]  mRNA expression and protein production in a time dependent and dose dependent manner in endothelial cells, decreased intracellular glutathione GSH and protein thiol levels, as well as G6PDH activity and NADPH levels.  Ref: 12883331 J Cardiovasc Pharmacol, 2003
- ELISA showed that the levels of MCP 1  [ **CCR2** ]   [ **CCL2** ]  protein in the conditioned media were also increased 2.9 fold, 1.7 fold and 1.1 fold, respectively.  Ref: 12845752 J Tongji Med Univ, 2000
- Adhesion induced expression of MCP 1  [ **CCR2** ]   [ **CCL2** ]  mRNA was inhibited by genistein and tosyl phenylalanyl chloromethylketone TPCK, suggesting that tyrosine kinases e.g., FAK, and nuclear factor kappa B NF kappaB are necessary in this signaling.  Ref: 12846738 Kidney Int, 2003
- The results were as follows Dot blotting analysis showed that the cultured SMCs could express MCP 1  [ **CCR2** ]   [ **CCL2** ]  mRNA.  Ref: 12845752 J Tongji Med Univ, 2000
- MCP 1  [ **CCR2** ]   [ **CCL2** ]  expression and production in monocyte macrophages from rats at normoxic conditions was reduced in aged subjects.  Ref: 12870648 Mol Cell Biochem, 2003
- The possible connection between periodontitis and atherosclerosis through IL 8 and MCP 1  [ **CCR2** ]   [ **CCL2** ]  is discussed.  Ref: 12887346 J Clin PeriodontolJ Clin Periodontol, 2003
- Additionally, resistin treated cells showed increased expression of VCAM 1 and MCP 1  [ **CCR2** ]   [ **CCL2** ] , with concomitant reductions in TRAF 3 expression.  Ref: 12874180 CirculationCirculation, 2003
- In pleural effusions, MCP 1  [ **CCR2** ]   [ **CCL2** ]  levels were significantly higher in patients with TBP than in patients with CHF, but lower than in malignant effusions.  Ref: 12861395 Inflamm Res, 2003
- Besides the staurosporines, also 12 O tetradecanoyl phorbol acetate TPA and tumor necrosis factor alpha TNFalpha strongly increased the IL 8 and MCP 1  [ **CCR2** ]   [ **CCL2** ]  secretion of NB 4 cells.  Ref: 12860017 Leuk Res, 2003
- The release of the chemokines interleukin IL 8 and macrophage chemoattractant protein MCP 1  [ **CCR2** ]   [ **CCL2** ]  in the cultures decreased twofold over time.  Ref: 12887346 J Clin PeriodontolJ Clin Periodontol, 2003
- After this time, total and lipoprotein cholesterol in the serum, hepatic tocopherol, aortic lesion area and MCP 1  [ **CCR2** ]   [ **CCL2** ]  protein and mRNA expression were analysed.  Ref: 12844369 Br J Nutr, 2003
- Expression patterns of MCP 1  [ **CCR2** ]   [ **CCL2** ]  and c fms exhibited no difference between the two lesion types.  Ref: 12892039 J Cardiol, 2003
- In situ hybridization showed that MCP 1  [ **CCR2** ]   [ **CCL2** ]  was located in corneal epithelial cells, and IL 1beta was located in corneal epithelial cells and infiltrating inflammatory cells.  Ref: 12861033 Lab Invest, 2003
- Therefore the current study investigated whether N acetylcysteine NAC, an antioxidative agent, inhibits the interleukin IL 1beta induced expression and production of eotaxin and monocyte chemotactic protein MCP 1  [ **CCR2** ]   [ **CCL2** ]  in human airway smooth muscle cells HASMC.  Ref: 12882449 Eur Respir J, 2003
- Several staurosporines increased, dose and time dependently, the IL 8 and MCP 1  [ **CCR2** ]   [ **CCL2** ]  concentration in the cell culture supernatants and three derivatives strongly inhibited proliferation of the NB 4 cells.  Ref: 12860017 Leuk Res, 2003
- These results demonstrate that homocysteine can trigger overexpression of the MCP 1  [ **CCR2** ]   [ **CCL2** ]  gene by altering the intracellular redox status, suggesting that the homocysteine induced changes in the intracellular redox status play an important role in modulating the expression of MCP  [ **CCL2** ]  l in endothelial cells.  Ref: 12883331 J Cardiovasc Pharmacol, 2003
- After a 4 h exposure to IL 1 beta, TNF alpha and LPS, the MCP 1  [ **CCR2** ]   [ **CCL2** ]  mRNA expression in SMCs was increased 3.6 fold, 2.3 fold and 1.6 fold, respectively.  Ref: 12845752 J Tongji Med Univ, 2000
- The MCP 1  [ **CCR2** ]   [ **CCL2** ]  protein content in the conditioned media was determined by using sandwich ELISA.  Ref: 12845752 J Tongji Med Univ, 2000
- CONCLUSION We postulate that TFC may play a role in the pathogenesis of autoimmune thyroid disease as they are able to express the chemokines MIP 1alpha, MIP 1beta, MCP 1  [ **CCR2** ]   [ **CCL2** ] , RANTES, IP 10 and Mig that would promote the infiltration of immune cells into the thyroid gland.  Ref: 12864798 Clin Endocrinol (Oxf), 2003
- Recent evidence from genetically modified mice indicates that MCP 1  [ **CCR2** ]   [ **CCL2** ]  and  **CCR2**  have unanticipated effects on T helper Th cell development.  Ref: 12851642 Microcirculation, 2003
- Using cultured endothelial cells, we have shown that laminar shear stress causes a transient increase in monocyte chemotactic protein 1  [ **CCL2** ]  MCP 1  [ **CCR2** ]   [ **CCL2** ]  expression, which involves the Ras MAP kinase signaling pathway.  Ref: 12865076 Prog Biophys Mol Biol, 2003
- At all the concentrations tested, MOM was more effective than DEX in inhibiting ICAM 1 expression and MCP 1  [ **CCR2** ]   [ **CCL2** ]  release p< ; 0.05, each comparison.  Ref: 12877820 Pulm Pharmacol Ther, 2003
- Objective The role of monocyte chemotactic protein MCP 1  [ **CCR2** ]   [ **CCL2** ]  in human pulmonary and pleural tuberculosis TB was assessed by examining its production in clinical samples from patients with active pulmonary TB and tuberculous pleurisy TBP.  Ref: 12861395 Inflamm Res, 2003
- However, unlike the identical phenotypes of MCP 1  [ **CCR2** ]   [ **CCL2** ]   **CCL2**  and  **CCR2**  mice in inflammatory diseases, the phenotypes of these mice are disparate in adaptive immunity MCP 1  [ **CCR2** ]   [ **CCL2** ]  stimulates Th2 polarization.  Ref: 12851642 Microcirculation, 2003
- The aim of the study was to evaluate the inhibitory effect of two corticosteroids, mometasone furoate MOM and dexamethasone DEX, respectively, on a variety of fibroblast functions DNA synthesis and proliferation, expression of adhesion molecules intercellular adhesion molecule 1 ICAM 1, CD54 and hyaluronic cellular adhesion molecule HCAM, CD44 and release of chemokines cytokines monocyte chemoattractant protein MCP 1  [ **CCR2** ]   [ **CCL2** ] , eotaxin, interleukin IL 6 and transforming growth factor TGF beta.  Ref: 12877820 Pulm Pharmacol Ther, 2003
- IL 8 and MCP 1  [ **CCR2** ]   [ **CCL2** ]  mRNA levels peaked within hours and were undetectable at 14 days.  Ref: 12866605 Vasc Med, 2003
- Infected VSMCs produced and released RANTES and MCP 1  [ **CCR2** ]   [ **CCL2** ] .  Ref: 12860900 CirculationCirculation, 2003
- When wild type and PAFR mice were compared, there were no marked changes in the kinetics of the expression of MCP 1  [ **CCR2** ]   [ **CCL2** ] , RANTES, IFN gamma and TNF alpha in heart tissue of infected animals.  Ref: 12850205 Microbes Infect, 2003
- We used a mouse inflammatory corneal model corneas cauterized with silver nitrate and assessed the role of monocyte macrophage attracting factors, macrophage chemotactic protein 1 MCP 1  [ **CCR2** ]   [ **CCL2** ] , and a proinflammatory cytokine, IL 1beta, on macrophage recruitment and neovascularization.  Ref: 12861033 Lab Invest, 2003
- There were positive correlations between the concentration of IL 8 and the percentage of neutrophils p = 0.038 and between the concentration of MCP 1  [ **CCR2** ]   [ **CCL2** ]  and the percentage of lymphocytes p = 0.006.  Ref: 12872499 G Ital Med Lav ErgonG Ital Med Lav Ergon, 2003
- We conclude that hyperoxia is an important regulator of MCP 1  [ **CCR2** ]   [ **CCL2** ]  release and support the hypothesis that increased % of O2 may serve to initiate MCP 1  [ **CCR2** ]   [ **CCL2** ]  production which then serves to recruit and regulate the distribution of mononuclear cells to the sites of inflammation.  Ref: 12870648 Mol Cell Biochem, 2003
- MgSO 4 inhibited the expression of Ps of platelets and MCP 1  [ **CCR2** ]   [ **CCL2** ]  mRNA of myocardial tissue and decreased serum Ca 2 concentration, but did not affect the expression of CD11b of leukocytes.  Ref: 12881868 Zhejiang Da Xue Xue Bao Yi Xue Ban, 2003
- The effects of resistin on expression of vascular cell adhesion molecule VCAM 1 and monocyte chemoattractant chemokine MCP 1  [ **CCR2** ]   [ **CCL2** ]  were studied in addition to CD40 receptor, CD40 ligand induced MCP 1  [ **CCR2** ]   [ **CCL2** ]  expression, and tumor necrosis factor receptor associated factor 3 TRAF3, an inhibitor of CD40 signaling.  Ref: 12874180 CirculationCirculation, 2003
- ACE Inhibitors Improve Diabetic Nephropathy Through Suppression of Renal MCP 1  [ **CCR2** ]   [ **CCL2** ] .  Ref: 12882873 Diabetes Care, 2003
- All integrin interacting substrates laminin and types I, III, and IV collagens also increased levels of FAK phosphorylation and MCP 1  [ **CCR2** ]   [ **CCL2** ]  expression.  Ref: 12846738 Kidney Int, 2003
- Plasma ET 1, ADMA, VCAM 1, and MCP 1  [ **CCR2** ]   [ **CCL2** ]  levels were significantly elevated in secondary RP compared with primary RP.  Ref: 12847693 Arthritis Rheum, 2003
- High levels of RANTES CCL5, MCP 1  [ **CCR2** ]   [ **CCL2** ]   **CCL2** , IP 10 CXCL10, and Mig CXCL9 simultaneously with mononuclear cell infiltration in the lungs was found.  Ref: 12875978 Am J Pathol, 2003
- We studied the effects of alpha tocopheryl acetate supplementation on the development of fatty streaks and its ability to modulate the expression of monocyte chemoattractant protein MCP 1  [ **CCR2** ]   [ **CCL2** ]  in aortic lesions of apoliprotein E knockout mice.  Ref: 12844369 Br J Nutr, 2003
- We studied polymorphisms in the genes for monocyte chemoattractant protein 1  [ **CCL2** ]  MCP 1  [ **CCR2** ]   [ **CCL2** ]  and CC chemokine receptor CCR 2  [ **CCR2** ]  in 171 Parkinson s disease PD patients and 340 controls.  Ref: 12889089 Mov Disord, 2003
- To investigate whether interleukin 1 beta IL 1 beta, tumor necrosis factor alpha TNF alpha and lipopolysaccharide LPS induce expression of monocyte chemoattractant protein 1  [ **CCL2** ]  MCP 1  [ **CCR2** ]   [ **CCL2** ]  mRNA and protein in calf aortic smooth muscle cells SMCs, calf aortic SMCs were cultured by a substrate attached explant method.  Ref: 12845752 J Tongji Med Univ, 2000
- This effect was also detected in a second PD group, suggesting a possible involvement of MCP 1  [ **CCR2** ]   [ **CCL2** ]  in PD.  Ref: 12889089 Mov Disord, 2003
- Both MCP 1  [ **CCR2** ]   [ **CCL2** ] , IL 1beta protein, and mRNA levels increased markedly 12 hours after the chemical cauterization.  Ref: 12861033 Lab Invest, 2003
- However, spontaneous, LPS or PHA induced MCP 1  [ **CCR2** ]   [ **CCL2** ]  production was up regulated by exposure to hyperoxic conditions in both young 62 9 02 8 pg ml, respectively and aged rats 79 12 17 10 pg ml, respectively.  Ref: 12870648 Mol Cell Biochem, 2003
- This presents both a challenge and an opportunity for targeting the MCP 1  [ **CCR2** ]   [ **CCL2** ]   **CCL2**   **CCR2**  axis in disease.Microcirculation 2003 1 47 257.  Ref: 12851642 Microcirculation, 2003
- increased CD40 ligand induced MCP 1  [ **CCR2** ]   [ **CCL2** ]  production.  Ref: 12874180 CirculationCirculation, 2003
- In the course of studying the effect of P. aeruginosa secretory factors on airway epithelial cells, we observed that metalloproteases in bacterial conditioned medium, as well as purified alkaline protease and elastase, degraded human RANTES, monocyte chemotactic protein 1  [ **CCL2** ]  MCP 1  [ **CCR2** ]   [ **CCL2** ] , and epithelial neutrophil activating protein 78 ENA 78.  Ref: 12859857 J Interferon Cytokine Res, 2003
- Effects of wild type and dominant negative FAK on MCP 1  [ **CCR2** ]   [ **CCL2** ]  expression were examined by a transient transfection assay.  Ref: 12846738 Kidney Int, 2003
- We detected an upregulation or de novo synthesis of interleukin 1alpha IL 1alpha, IL 1beta, IL 8, macrophage inflammatory protein 1alpha MIP 1alpha, MIP 1beta, GRO alpha, transforming growth factor beta, MCP 1  [ **CCR2** ]   [ **CCL2** ] , IL 2 receptor gamma IL 2Rgamma, IL 10Ralpha, and IL 6R.  Ref: 12874345 Infect Immun, 2003
- Mounting evidence suggests that  **CCL2**  MCP 1  [ **CCR2** ]   [ **CCL2** ]  and its hematopoietic cell receptor CC chemokine receptor 2  [ **CCR2** ]   **CCR2**  are involved in inflammatory disorders of the lung.  Ref: 12851645 Microcirculation, 2003
- The molecular signals responsible for this directed migration are incompletely understood, but members of the chemokine family, especially the monocyte chemoattractant proteins MCPs MCP 1  [ **CCR2** ]   [ **CCL2** ]  to MCP  [ **CCL2** ]  5 are emerging as key players.  Ref: 12851643 Microcirculation, 2003
- Overexpression of wild type FAK increased phosphorylation of FAK and expression of MCP 1  [ **CCR2** ]   [ **CCL2** ]  mRNA and protein.  Ref: 12846738 Kidney Int, 2003
- Thus, Ado increases the release of IL 6 and MCP 1  [ **CCR2** ]   [ **CCL2** ]  from BSMCs via activation of the A2B AdoR.  Ref: 12855406 Am J Respir Cell Mol Biol< MedlineTA>Am J Respir Cell Mol Biol, 2003
- In conclusion, the results obtained here are relevant to the study of atherosclerosis, as they correlate the effectiveness of vitamin E supplementation in inhibiting the plaque formation with diminished expression of MCP 1  [ **CCR2** ]   [ **CCL2** ]  at the aortic lesion.  Ref: 12844369 Br J Nutr, 2003
- The results suggest that calf aortic SMCs could express MCP 1  [ **CCR2** ]   [ **CCL2** ]  mRNA and protein.  Ref: 12845752 J Tongji Med Univ, 2000
- Recent studies have demonstrated that SMC possess a number of functional chemokine receptors, including CCR5, CXCR4, and a receptor for monocyte chemoattractant protein 1  [ **CCL2** ]  MCP 1  [ **CCR2** ]   [ **CCL2** ] .  Ref: 12851644 Microcirculation, 2003
- Monocyte chemoattractant protein 1  [ **CCL2** ]  MCP 1  [ **CCR2** ]   [ **CCL2** ]  and interleukin 8 IL 8 are major chemokines for leukocyte trafficking and have been identified in atheromatous plaques.  Ref: 12881478 Circ ResCirc Res, 2003
- NECA increased the release of IL 6 and MCP 1  [ **CCR2** ]   [ **CCL2** ]  proteins with EC50 values of 1.26 0.25 micro M and 0.40 0.08 micro M, respectively, and the maximal folds of induction were 20.8 1.7 and 6.4 0.7 fold, respectively.  Ref: 12855406 Am J Respir Cell Mol Biol< MedlineTA>Am J Respir Cell Mol Biol, 2003
- In contrast, administration of the anti MCP 1  [ **CCR2** ]   [ **CCL2** ]  or anti IL 1beta showed minimal inhibition of basic fibroblast growth factor driven corneal neovascularization by mouse cornea assay.  Ref: 12861033 Lab Invest, 2003
- Although no associations were found in alleles or genotypes, MCP 1  [ **CCR2** ]   [ **CCL2** ]  2518A G genotype affected the age at onset of PD patients.  Ref: 12889089 Mov Disord, 2003
- The MCP 1  [ **CCR2** ]   [ **CCL2** ]  protein level in the cortical ischemic cores of the normothermic subgroups began to increase since the 6th hour afer reperfusion 22.5 8.7 ng.g tissue 7 times that in the sham operation samples, P <.  Ref: 12887739 Zhonghua Yi Xue Za Zhi, 2003
- These results indicate the existence of distinct monocyte macrophage involved angiogenic pathways in mouse cornea, in which MCP 1  [ **CCR2** ]   [ **CCL2** ]  released from corneal epithelial cells attracts monocytes macrophages into the cornea, where they release IL 1beta leading to inflammatory neovascularization.  Ref: 12861033 Lab Invest, 2003
- Sustained shear stress downregulates atherogenic genes e.g., MCP 1  [ **CCR2** ]   [ **CCL2** ]  and the genes that facilitate lipid accumulation and upregulates growth arrest genes.  Ref: 12865076 Prog Biophys Mol Biol, 2003
- Both IL 1beta and MCP 1  [ **CCR2** ]   [ **CCL2** ]  induced neovascularization in a rat cornea model, and the cauterization induced corneal neovascularization was partially inhibited by subconjunctival injection of anti IL 1beta or anti MCP 1  [ **CCR2** ]   [ **CCL2** ] .  Ref: 12861033 Lab Invest, 2003
- CONCLUSION Mild hypothermia reduces the level of MCP 1  [ **CCR2** ]   [ **CCL2** ]  in the cortex after cerebral ischemia reperfusion which may be one of the important mechanisms of the neuroprotective effects of mild hypothermia.  Ref: 12887739 Zhonghua Yi Xue Za Zhi, 2003
- ... TNF alpha induced a significant upregulation in ICAM 1 expression and in MCP 1  [ **CCR2** ]   [ **CCL2** ] , eotaxin and IL 6 release p< ; 0.05, each comparison.  Ref: 12877820 Pulm Pharmacol Ther, 2003
- NAC 10 mM decreased the expression of eotaxin and MCP 1  [ **CCR2** ]   [ **CCL2** ] , by 46 11% n=7 and 87 4% n=6, respectively.  Ref: 12882449 Eur Respir J, 2003
- We evaluated nine naturally occurring staurosporine derivatives as modulators of chemokine production by monitoring the secretion of interleukin 8 IL 8 and monocyte chemotactic protein 1  [ **CCL2** ]  MCP 1  [ **CCR2** ]   [ **CCL2** ]  in the cell line NB 4.  Ref: 12860017 Leuk Res, 2003
- In a mouse macrophage like cell line, the same beta isoform activation increases the release of the MCP 1  [ **CCR2** ]   [ **CCL2** ]  chemokine.  Ref: 12892998 Mol Aspects MedMol Aspects Med, 2003
- However, the M CSF c fms system, rather than MCP 1  [ **CCR2** ]   [ **CCL2** ] , is more important in the late stage of restenosis.  Ref: 12892039 J Cardiol, 2003
- CONCLUSION On the basis of previous studies and the present case, the high production of IL 8 and MCP 1  [ **CCR2** ]   [ **CCL2** ]  by monocytes in LPS stimulated WBCC from periodontitis patients is most likely acquired, as their levels decrease over time when the periodontal infection is controlled.  Ref: 12887346 J Clin PeriodontolJ Clin Periodontol, 2003
- We examined if blockade of the renin angiotensin system lowered urinary levels of the chemokine MCP 1  [ **CCR2** ]   [ **CCL2** ]  and correlated urinary MCP 1  [ **CCR2** ]   [ **CCL2** ]  uMCP 1 with parameters of renal function and glucose and lipid metabolism before and after 1 year of treatment with an ACE inhibitor.  Ref: 12882873 Diabetes Care, 2003
- Twenty five subjects 24 males and 1 female, mean age 57.4 years who have been exposed to asbestos underwent chest radiography, high resolution computed tomography HRCT of the chest, lung function tests and bronchoalveolar lavage BAL for evaluation of cell components total cell count, percentages of macrophages, lymphocytes, neutrophil and eosinophil granulocytes and the lymphocyte subpopulations CD3, CD4, CD8, CD19 and HLADR, soluble factors IL 8, IL 10, IL 12 and MCP 1  [ **CCR2** ]   [ **CCL2** ]  in the supernatant and concentration of asbestos fibre.  Ref: 12872499 G Ital Med Lav ErgonG Ital Med Lav Ergon, 2003
- OBJECTIVE To investigate the effect of intraischemic mild hypothermia on the protein levels of interleukin IL 1beta and monocyte chemoattractant protein MCP 1  [ **CCR2** ]   [ **CCL2** ]  in the ischemic core of rat cortex after transient focal cerebralischemia.  Ref: 12887739 Zhonghua Yi Xue Za Zhi, 2003
- The expression of MCP 1  [ **CCR2** ]   [ **CCL2** ]  mRNA in SMCs was detected by using dot blotting analysis using a probe of gamma 32P end labelled 35 mer oligonucleotide.  Ref: 12845752 J Tongji Med Univ, 2000
- the expression of CD11b of leukocytes was significantly higher than that of the control groups P< ; 0.01 and P< ; 0.05 the expression of MCP 1  [ **CCR2** ]   [ **CCL2** ]  mRNA of myocardial tissue began to rise at R60, reaching the highest at R360 P< ; 0.01 the concentration of Ca 2 of the serum was significantly higher than that of the control groups P< ; 0.01 and P< ; 0.05.  Ref: 12881868 Zhejiang Da Xue Xue Bao Yi Xue Ban, 2003
- ... the MCP 1  [ **CCR2** ]   [ **CCL2** ]  release was decreased by 69 41% n=10.  Ref: 12882449 Eur Respir J, 2003
- The following primary antibodies were used anti MCP 1  [ **CCR2** ]   [ **CCL2** ] , anti c fms, anti macrophages and anti alpha smooth muscle actin.  Ref: 12892039 J Cardiol, 2003
- , MCP 1  [ **CCR2** ]   [ **CCL2** ] , FGF 2, TGF beta, VEGF, and GM CSF.  Ref: 12851653 Microcirculation, 2003
- We also observed a significant decrease in the expression and the release of eotaxin, MCP 1  [ **CCR2** ]   [ **CCL2** ]  and MCP  [ **CCL2** ]  3 in the presence of SB203580, an inhibitor of p38 MAPK 71 6%, P <.  Ref: 12854631 Respir Med, 2003
- MgSO 4 may protect myocardial tissue from ischemic reperfusion injury by inhibiting the expression of Ps of platelets and MCP 1  [ **CCR2** ]   [ **CCL2** ]  mRNA of myocardial tissue and decreasing the concentration of Ca 2 of the serum.  Ref: 12881868 Zhejiang Da Xue Xue Bao Yi Xue Ban, 2003
- RESULTS In sera, the MCP 1  [ **CCR2** ]   [ **CCL2** ]  levels of TB patients were similar to those of HTRs.  Ref: 12861395 Inflamm Res, 2003
- In addition, the IL 1beta and MCP 1  [ **CCR2** ]   [ **CCL2** ]  released from the corneal epithelial cells may directly induce corneal neovascularization.  Ref: 12861033 Lab Invest, 2003
- We conclude that p38 MAPK, JNK kinase, ERK and NF kappaB are involved in the IL 1beta induced eotaxin, MCP 1  [ **CCR2** ]   [ **CCL2** ] , and MCP  [ **CCL2** ]  3 expression and release in HASMC.  Ref: 12854631 Respir Med, 2003
- Furthermore, the inhibitors of mitogen activated protein kinase p38 and extracellular signal regulated kinase 1 2 and nuclear factor kappaB or the activator of peroxisome proliferator activated receptor gamma PPARgamma significantly decreased Hcy induced MCP 1  [ **CCR2** ]   [ **CCL2** ]  and IL 8 secretion in these cells.  Ref: 12881478 Circ ResCirc Res, 2003
- MCP 1  [ **CCR2** ]   [ **CCL2** ]  and IL 8 have been found to express mainly by macrophages in human lesion.  Ref: 12881478 Circ ResCirc Res, 2003
- Interestingly, the E TB patients had significantly depressed MCP 1  [ **CCR2** ]   [ **CCL2** ]  production by PBMCs in response to PPD or 30 kDa, compared with HTRs and CR TB patients.  Ref: 12861395 Inflamm Res, 2003
- We examined the effects of increased extracellular matrix ECM accumulation on monocyte chemoattractant protein 1  [ **CCL2** ]  MCP 1  [ **CCR2** ]   [ **CCL2** ]  expression, which is known to play an important role in the progression of various glomerular diseases.  Ref: 12846738 Kidney Int, 2003
- These data indicate that pathophysiological levels of Hcy can alter human monocyte function by upregulating MCP 1  [ **CCR2** ]   [ **CCL2** ]  and IL 8 expression and secretion via enhanced formation of intracellular ROS originated from NAD P H oxidase source via calmodulin or protein kinase C signaling pathways.  Ref: 12881478 Circ ResCirc Res, 2003
- The protein level of MCP 1  [ **CCR2** ]   [ **CCL2** ]  in the mild hypothermic subgroups was 8.7 7.6 ng.g tissue 1 6 h after reperfusion P <.  Ref: 12887739 Zhonghua Yi Xue Za Zhi, 2003
- The expression of Ps of platelets and CD11b of leukocytes was determined by flowcytometry and the expression of MCP 1  [ **CCR2** ]   [ **CCL2** ]  mRNA of myocardial tissue was measured by RT PCR admission and at 8 60 min of reperfusion R5, R30, R60, R180, R360.  Ref: 12881868 Zhejiang Da Xue Xue Bao Yi Xue Ban, 2003
- These effects of Hcy were primarily mediated by reactive oxygen species ROS through NAD P H oxidase, because Hcy could upregulate the production of ROS and the inhibitors of protein kinase C, calmodulin, free radical scavengers, or NAD P H oxidase abolished Hcy induced ROS production and MCP 1  [ **CCR2** ]   [ **CCL2** ]  and IL 8 secretion in these cells.  Ref: 12881478 Circ ResCirc Res, 2003
- Angiotensin II induces several fibrogenic chemokines, namely monocyte chemoattractant protein 1  [ **CCL2** ]  MCP 1  [ **CCR2** ]   [ **CCL2** ]  and transforming growth factor beta.  Ref: 12882873 Diabetes Care, 2003
- The expression of MCP 1  [ **CCR2** ]   [ **CCL2** ]  positive cells almost coincided with the macrophages.  Ref: 12892039 J Cardiol, 2003
- The protein levels of IL 1beta and MCP 1  [ **CCR2** ]   [ **CCL2** ]  in the cortical ischemic core were measured by ELISA.  Ref: 12887739 Zhonghua Yi Xue Za Zhi, 2003
- In contrast, staining for MCP 1  [ **CCR2** ]   [ **CCL2** ]  was little seen in the stellate vascular smooth muscle cells.  Ref: 12892039 J Cardiol, 2003
- In contrast, disturbed flow observed at branch points and simulated in step flow channels causes sustained activation of MCP 1  [ **CCR2** ]   [ **CCL2** ]  and the genes facilitating cell turnover and lipid accumulation.  Ref: 12865076 Prog Biophys Mol Biol, 2003
- Urinary MCP 1  [ **CCR2** ]   [ **CCL2** ]  levels decreased from 0.456 0.22 ng mg creatinine to 0.08 0.096 ng mg creatinine.  Ref: 12882873 Diabetes Care, 2003
- Oxygen supply modulates MCP 1  [ **CCR2** ]   [ **CCL2** ]  release in monocytes from young and aged rats decrease of MCP 1  [ **CCR2** ]   [ **CCL2** ]  transcription and translation is age related.  Ref: 12870648 Mol Cell Biochem, 2003
- The inhibition of PNF by AsA was associated with decreased intra islet levels of inflammation related molecules IL 1, TNF alpha, iNOS, COX 2 and chemokines MCP 1  [ **CCR2** ]   [ **CCL2** ]  and MIP 3alpha.  Ref: 12879250 DiabetologiaDiabetologia, 2003
- Monocyte chemoattractant protein MCP 1  [ **CCR2** ]   [ **CCL2** ]   **CCL2**  specifically attracts monocytes and memory T cells.  Ref: 12851642 Microcirculation, 2003
- In this study we evaluated if the ability of monocytes to produce MCP 1  [ **CCR2** ]   [ **CCL2** ]  under basal conditions or after stimulation with lipopolysaccharide LPS or phytohaemagglutinin PHA was differently affected by exposure to hyper or hypoxic conditions in young and aged rats.  Ref: 12870648 Mol Cell Biochem, 2003
- In parallel, the response to  **CCL2**  MCP 1  [ **CCR2** ]   [ **CCL2** ]  was lost after infection with active virus Neither a  **CCL2**  MCP 1  [ **CCR2** ]   [ **CCL2** ]  induced shift of intracellular calcium concentrations nor the chemotactic response to  **CCL2**  MCP 1  [ **CCR2** ]   [ **CCL2** ]  was detectable.  Ref: 12885942 J Leukoc Biol, 2003
- OBJECTIVE To investigate the effect of ischemic reperfusion I R on the expression of P selectin Ps of platelets, CD11b of leukocytes, monocyte chemotactic protein 1  [ **CCL2** ]  MCP 1  [ **CCR2** ]   [ **CCL2** ]  mRNA of myocardial tissue and the protective role of MgSO 4.  Ref: 12881868 Zhejiang Da Xue Xue Bao Yi Xue Ban, 2003
- We demonstrated the spatial pattern of MCP 1  [ **CCR2** ]   [ **CCL2** ]  mRNA levels through in situ mRNA hybridization.  Ref: 12866605 Vasc Med, 2003
- Metalloproteases from Pseudomonas aeruginosa degrade human RANTES, MCP 1  [ **CCR2** ]   [ **CCL2** ] , and ENA 78.  Ref: 12859857 J Interferon Cytokine Res, 2003
- The early response was characterized by high levels of inflammatory molecules, including IL 1beta, IL 6, TNFalpha, RANTES, MIP 1alpha and MCP 1  [ **CCR2** ]   [ **CCL2** ] , later followed by expression of precursor Th1 cytokines, IL 12 and IL 18, concomitant with IFNgamma production.  Ref: 12850812 Cytokines Cell Mol Ther, 2003
- IL 1 beta and TNF alpha can induce strong expression of MCP 1  [ **CCR2** ]   [ **CCL2** ]  mRNA and protein, and the former is more effective than the latter.  Ref: 12845752 J Tongji Med Univ, 2000
- RESULTS Expression of MCP 1  [ **CCR2** ]   [ **CCL2** ] , RANTES, MIP 1alpha, MIP 1beta, IP 10 and Mig was demonstrated in all Hashimoto s and most Graves thyroid specimens but very little expression was detected in the nonautoimmune goitre samples.  Ref: 12864798 Clin Endocrinol (Oxf), 2003
- CONCLUSIONS Both MCP 1  [ **CCR2** ]   [ **CCL2** ]  and the M CSF c fms system are involved in the atherogenesis of de novo lesions.  Ref: 12892039 J Cardiol, 2003
- Neutralizing antibodies against human RANTES, MIP 1alpha, MIP 1beta, alpha interferon IFN alpha, IFN beta, IFN gamma, interleukin 4 IL 4, IL 10, IL 13, IL 16, MCP 1  [ **CCR2** ]   [ **CCL2** ] , MCP  [ **CCL2** ]  3, tumor necrosis factor alpha TNF alpha, or TNF beta failed to reverse the HIV 1 suppressive activity.  Ref: 12885891 J VirolJ Virol, 2003
- Up regulation of IL 6, IL 8, and MCP 1  [ **CCR2** ]   [ **CCL2** ]  mRNA expression and production by the virus in combination with opaque S. pneumoniae was two to threefold higher than that induced by the virus combined with the transparent S. pneumoniae variant.  Ref: 12874304 Infect Immun, 2003
- However, both S. pneumoniae opacity variants increased mRNA expression of MIP 1alpha, MIP 1beta, IL 6, and MCP 1  [ **CCR2** ]   [ **CCL2** ]  in HMEE cells activated by a prior influenza A virus infection compared to levels in cells treated with either agent alone.  Ref: 12874304 Infect Immun, 2003
- Asthma is an inflammatory disease, in which eotaxin, MCP 1  [ **CCR2** ]   [ **CCL2** ]  and MCP  [ **CCL2** ]  3 play a crucial role.  Ref: 12854631 Respir Med, 2003
- We analyzed the mRNA levels of the chemokines monocyte chemoattractant protein 1  [ **CCL2** ]  MCP 1  [ **CCR2** ]   [ **CCL2** ]  and interleukin 8 IL 8 through semi quantitative polymerase chain reaction.  Ref: 12866605 Vasc Med, 2003
- IL 8 and MCP 1  [ **CCR2** ]   [ **CCL2** ]  mRNA levels peaked within hours but were still detectable 14 days post injury.  Ref: 12866605 Vasc Med, 2003
